# Cytokines in tear fluid of patients with ocular graft-versus-host disease after allogeneic stem cell transplantation

**Published:** 2012-04-01

**Authors:** Anjo Riemens, Elena Stoyanova, Aniki Rothova, Jonas Kuiper

**Affiliations:** 1 Department of Ophthalmology, Medical Center Utrecht, Utrecht, the Netherlands; 2 Department of Ophthalmology, Erasmus Medical Center Rotterdam, Utrecht, the Netherlands; 3 Department of Hematology, University Medical Center Utrecht, Utrecht, the Netherlands

## Abstract

**Purpose:**

To investigate the profile of cytokines in tear fluid of patients after allogeneic stem cell transplantation (allo-SCT) and determine their relation to the presence and manifestations of ocular graft-versus-host disease (GvHD).

**Methods:**

In this cross sectional study tear fluid was collected in 34 consecutive adult patients that previously underwent allo-SCT (16 with ocular GvHD and 18 without) and 16 age- and gender-matched healthy controls using the Schirmer test under local anesthesia. Tear fluid was analyzed by multiplex immunoassay for the presence of interleukin (IL)-2, IL-4, IL-6, IL-10, IL-17, tumor necrosis factor (TNF)-α and interferon (IFN)-γ. Levels of measured cytokines were correlated with the findings in slit lamp examination and the Ocular Surface Disease Index (OSDI).

**Results:**

The levels of IL-6 and IFN-γ in tear fluid in ocular GvHD patients were significantly elevated in comparison to patients without ocular GvHD and healthy controls (p<0.005 for each) The levels of IFN-γ correlated with the Schirmer score (r=-0.48, p<0.0001) and tear break up time (TBUT; r=-0.38, p=0.03). Tear IL-6 levels correlated with complaints of dry eyes (r=0.39, p=0.02), tear production (r=-0.59, p<0.0001), fluorescent staining of the cornea (r=0.42, p=0.01), and with the OSDI score (r=0.40, p=0.005).

**Conclusions:**

IL-6 and IFN-γ were elevated in tear fluid of patients with ocular GvHD and correlated with different symptoms of dry eye disease, suggesting that IFN-γ is elevated during the early stages and IL-6 is involved in later stages of ocular GVHD and exhibits moreover an association with its severity.

## Introduction

Hematopoietic stem cell transplantation (SCT) is the treatment of choice for many life-threatening malignant and non-malignant hematologic diseases. Allogeneic SCT (allo-SCT) is commonly accompanied by graft-versus-host disease (GvHD), a multi-organ systemic disease associated with high morbidity and mortality. Ocular GvHD predominantly affects the anterior ocular segment and manifestations include conjunctival or corneal epithelial changes, Meibomian gland dysfunction and dry eye disease (DED) manifesting as keratoconjunctivitis sicca (KCS) [1,2]. KCS is the most frequently occurring symptom of ocular GvHD and until now its presence is used to diagnose ocular GvHD [3]. The pathogenesis of ocular GvHD has not yet been clarified. Cytokines have been suggested to play a major role in development of systemic and ocular GvHD and increased levels of interleukin (IL)-6 and interferon gamma (IFN-γ) have been observed in serum in patients with systemic acute GvHD [4].

Increased levels of pro-inflammatory cytokines in tear fluid, including interleukin (IL)-6 and IFN-γ were noted in DED of various origins, nonetheless their role in DED is not yet clarified [5,6].

The purpose of this study was to determine the cytokine levels in tear fluid in patients after allo-SCT and to identify their relation to the presence of ocular GvHD and clinical symptoms of DED.

## Methods

The study was performed in accordance with the Declaration of Helsinki and with the approval of the local Institutional Review Board.

### Patients

In this cross-sectional pilot study, we included 34 consecutive allo-SCT patients referred to the Department of Ophthalmology of the University Medical Centre of Utrecht (UMCU). Patients with ocular comorbidity and patients with a mean Schirmer of ≤1 mm in 5 min were excluded (n=1). Patients consulted our ophthalmology clinic 3 months after SCT for screening or at any other time if they had ocular complaints after SCT. Sixteen gender- and age-matched healthy volunteers with no history of ocular disease, current systemic and ocular infection were included as controls.

### Diagnostic criteria

All patients underwent a visual acuity test, slit-lamp examination and a Schirmer test as part of the standard examination for ocular GvHD. The diagnosis of ocular GvHD was based on the National Institute for Health (NIH) consensus criteria, which combines the presence of systemic GvHD manifestations with either a mean Schirmer test value ≤5 mm in 5 min or newly developed slit-lamp-confirmed KCS with mean score of Schirmer test between 6 and 10 mm in 5 min (with local anesthesia [Oxybuprocain 0.4%]) [3]. The diagnosis of KCS with the slit-lamp was made based on the presence of fluorescent staining of the cornea, decreased tear break up time (TBUT) and complaints of dry eyes such as grittiness and blurring of vision. According to NIH consensus criteria [3], blepharitis, Meibomian gland dysfunction, and chemosis were not taken into account for the diagnosis of ocular GvHD. Patients and controls completed the Ocular Surface Disease Index (OSDI) questionnaire to determine the subjective ocular symptoms as described previously [7,8].

### Tear sample collection

The tear production was determined with a Schirmer test under local anesthetic drops (Oxybuprocaine HCl, 0.4%) applied 3 min before applying the strip. The Schirmer strip (Schirmer Tear Test Strips; Biotech Vision Care Pvt. Ltd., Ahmedabad, Gujarat, India) was placed in the lateral lower conjunctival sac and the participants were instructed to close their eyes. After 5 min the strips were removed and the tear production recorded in millimeters. Each Schirmer strip was immediately placed in an Eppendorf plastic tube (Sarstedt, Numbrecht, Germany) and diluted 1:20 with phosphate-buffered saline solution (PBS, pH=7.4). The strips were incubated at 4 °C overnight and stored at –80 °C until use.

### Cytokine concentration analysis

The tear fluid of the eye with the lowest Schirmer score of each subject was analyzed. If the values in both eyes were equal, the Schirmer strip of the right eye was chosen for analysis. The levels of IL-2, IL-4, IL-6, IL-10, IL-17A, TNF-α, and IFN-γ in tears were measured by multiplex immunoassay (Cytometric Bead Array; BD Biosciences, San Jose, CA) according to the manufacturer’s protocol. Detection limits for IL-2, IL-4, IL-6, IL-10, IL-17A, TNF-α, and IFN-γ were 2.6, 4.9, 2.4, 4.5, 18.9, 3.8, and 3.7 pg/ml, respectively. Concentrations were calculated from the generated standard curves.

### Statistical analysis

Statistical analysis was performed with the SPSS software package (SPSS version 15 for Windows; Chicago, IL). Analysis of variance (ANOVA), Mann–Whitney U test, and Fisher’s exact test were used to analyze group differences. Correlation between clinical parameters and interleukin levels was determined by Spearman rank test. P-values were adjusted for multiple comparisons using a Bonferroni’s correction. A p-value less than 0.05 was regarded statistically significant.

## Results

### Study population

Study group demographic details are summarized in Table 1; 34 patients after allo-SCT, 16 with ocular GvHD and 18 patients without ocular GvHD were included. The healthy controls (n=16; mean age: 48.0±15.1, male/female ratio: 11/5) were age- and gender- matched to the patients group. The median interval time between the last allo-SCT and clinical examination was 37.2 months (interquartile range [iq] range 68) for the ocular GvHD group and 3 months (iq range 3) for the no ocular GvHD group (p=0.007).

**Table 1:** General characteristics of patients after allogeneic stem cell transplantation (allo-SCT).

**Patient characteristics**	**Total, allo-SCT patients (n=34)**	Ocular GvHD* (n=16)	**No Ocular GvHD (n=18)**
Mean age (yrs)±SD (Minimum, maximum)	51.5±13.8 (18–71)	50.1±15.8 (23–71)	52.7±12.1 (18–64)
**Gender, n (%)**			
Male	22/34 (65%)	13/16 (81%)	9/18 (50%)
Female	12/34 (35%)	3/16 (19%)	9/18 (50%)
**Follow-up last transplantation, n (%)**			
≤100 days	15/34 (44%)	3/16 (19%)	12/18 (67%)
>100 days	19/34 (56%)	13/16 (81%)	6/18 (33%)
**Type of transplant, n (%)**			
Matched related donor	11/34 (32%)	9/16 (56%)	2/18 (11%)
Matched unrelated donor	23/34 (68%)	7/16 (44%)	16/18 (89%)
**TBI, n (%)**			
Yes	27/34 (79%)	13/16 (81%)	14/18 (78%)
Unknown	2/34 (6%)	1/16 (6%)	1/18 (6%)
**TBI and chemotherapy (ATG or FLU)**			
Yes	27/34 (54%)	13/16 (81%)	14/18 (78%)
Unknown	2/34 (4%)	1/16 (6%)	1/18 (6%)
**Type of disorder, n (%)**			
Multiple myeloma	10/34 (29%)	5/16 (31%)	5/18 (28%)
AML	9/34 (26%)	1/16 (6%)	8/18 (44%)
NHL	5/34 (15%)	3/16 (19%)	2/18 (11%)
ALL	3/34 (9%)	1/16 (6%)	2/18 (11%)
CLL	1/34 (3%)	1/16 (6%)	0/18 (0%)
HL	1/34 (3%)	1/16 (6%)	0/18 (0%)
CML	1/34 (3%)	1/16 (6%)	0/18 (0%)
Other	4/34 (12%)	3/16 (19%)	1/18 (6%)

n, Number of patients; FLU, Fludarabine; CML, Chronic myelcytic leukemia; ATG, Anti-thymocyte globulin; ALL, Acute lymphblastic leukemia; Allo-SCT, allogeneic stem cell transplantation; AML, Acute myeloblastic leukemia; CLL, Chronic lymphocytic leukemia; GvHD, Graft-verus-Host-Disease; HL, Hodgkin’s lymphoma; NHL, non-Hodgkin’s lymphoma; SD, standard deviation; TBI, Total body irradiation. *diagnosed according to the National Institute for Health consensus criteria [3].

### Diagnostic clinical tests

Of all patients with ocular GvHD 14/16 (88%) had complaints of dry eyes, 6/16 (38%) had a decreased TBUT and in 9/16 (56%) fluorescein staining of the cornea was seen.

The mean Schirmer score was lower in the ocular GvHD group (4.4±2.0 mm) in comparison to the no ocular GvHD group (16.2±7.5 mm, p<0.0001) and the healthy control group (22.1±7, p<0.0001). The median OSDI score was significantly impaired in the ocular GvHD compared to the two other study groups (Table 2).

**Table 2:** Inflammatory cytokines in tear fluid and ocular findings after allo-ACT.

**Inflammatory cytokines and ocular findings**	**Allo-SCT patients (n=34)**	**Ocular GvHD patients (n=16)**	**No ocular GvHD patients (n=18)**	**Healthy Controls (n=16)**	**p-value, Ocular GvHD versus No Ocular GvHD**	**p-value, Ocular GvHD versus Healthy Controls**	**p-value, No Ocular GvHD versus Healthy Controls**
IL-6, pg/ml, n (%)	15/34 (44%)	11/16 (69%)	4/18 (22%)	1/16 (6%)	p=0.005	p<0.0001	NS
Median, iq range	0, 130.6	107.4, 417.1	0, 7.9	0, 0			
IFN-γ, pg/ml, n (%)	8/34 (23%)	8/16 (50%)	0/18 (0%)	0/16 (0%)	p=0.001	p=0.002	NA
Median, iq range	0, 0	572, 1372.9	0, 0				
Mean Schirmer ±SD, mm	10.7±8.2 mm	4.4±2.0 mm	16.2±7.5 mm	22.1±7 mm	p<0.0001	p<0.0001	p=0.021
Median OSDI score, iq range	11.1, 14.0	13.9, 21.2^1)^	6.0, 27.8^2)^	0, 3.7	p=0.015	p<0.0001	p=0.026

Allo-SCT, allogeneic stem cell transplantation; n, Number of patients; GvHD, Graft-versus-Host-Disease; IFN- γ, interferon- γ; IL-6, interleukin-6; Iq range, Interquartile range; OSDI, Ocular Surface Disease Index; NS=Not statistically significant NA, Not Applicable; SD, Standard Deviation; ^1)^ Data from 1 patient is missing; ^2)^ Data from 2 patients is missing.

### Cytokine tear levels

Results of the tear cytokine levels are shown in Table 2. IL-6 was detected in the tear fluid of 15/34 (44%) allo-SCT patients and in 1/16 (6%) of healthy controls (p<0.0001). The median level of IL-6 in tear fluid was significantly elevated in the ocular GvHD group compared to the no ocular GvHD group (p=0.005), and the control group (p<0.0001). IL-6 levels in patients without ocular GvHD patients and healthy controls did not differ (p=0.162). IFN-γ was detected in tear fluid in 8/16 (50%) ocular GvHD patients. IFN-γ could not be detected in the other groups. IL-2, IL-4, IL-10, IL-17, and TNF-α could not be detected in any of the samples. The mean age of patients in which IL-6 (53.8±13) or IFN-γ (53.9±12) was detected, was not different from patients in whom no IL-6 (48.5±14) or IFN-γ (43.6±16) could be detected (p=0.28; p=0.11).

### Correlation between cytokines and clinical manifestations of ocular GVHD

Correlation analysis was performed between tear cytokine levels and symptoms of ocular GvHD for IL-6 and IFN-γ. No significant correlation could be found between the levels of IFN- γ and IL-6 (r=0.23, p=0.189).Tear IL-6 levels correlated with complaints of dry eyes (r=0.39, p=0.02), tear production measured by Schirmer test (r=-0.59, p<0.0001), fluorescent staining of the cornea (r=0.42, p=0.01), and OSDI score (r=0.40, p=0.005). IL-6 did not correlate with TBUT (r=-0.03, p=0.88). The levels of IFN-γ correlated with the tear production measured by Schirmer test (r=-0.48, p<0.0001) and TBUT (r=-0.38, p=0.03). However, IFN-γ did not correlate significantly with complaints of dry eyes (r=0.12, p=0.48), fluorescent staining of the cornea (r=0.10, p=0.57), or OSDI scores (r=0.16, p=0.61).

## Discussion

In this study, significantly elevated IL-6 and IFN-γ levels in tear fluid in patients with ocular GvHD were detected. These cytokines were associated with different clinical features: IL-6 with complaints of dry eyes, OSDI scores and corneal staining whereas IFN-γ showed an association with decreased TBUT and low Schirmer test score. These results might demonstrate that IFN-y is elevated in the early stage of ocular GvHD and that IL-6 is elevated later in the disease and is associated with the severity of ocular GvHD.

The measurements of tear production using Schirmer test might reveal variable results [9]. When applying the strips without previous anesthesia, the results are a subject to variability due to reflex tearing and when using prior anesthesia, the residual amount of topical anesthetics might influence the Schirmer values. To minimize these variable measurements, we have chosen to apply anesthetic drops to measure a basal tear production and further included a standard interval between the application of anesthetic drops and Schirmer test to minimize the influence of their possible residual presence.

IFN-γ is a T-cell associated cytokine that plays a major role in the pathogenesis of GvHD [10]. IL-6 is known to contribute to GvHD severity [11] but has also been reported to be correlated with disease onset [12].

The current knowledge about the involvement of interleukins in ocular GvHD is very limited. The conjunctiva is known to be one of the target organs in the T-cell mediated GvHD, resembling complex autoimmune fibrotic processes [13-15].

Ogawa et al. [13] reported that donor-derived cluster of differentiation (CD)34+ stromal fibroblasts were present in the lacrimal gland of DED patients after allo-SCT. Subsets of stromal fibroblasts can function as antigen presenting cells and activate donor T-cells, which may result in the formation of pro-inflammatory cytokines like IL-6 and IFN-γ [13].

Elevated levels of IL-6 and IFN-γ in tears were previously found and were associated with DED seen in different etiologies, including Sjorgren’s disease [5,16,17]. IFN-γ is a crucial cytokine in the induction of GvHD [4]. Recently, a central role for IFN-γ-producing natural killer cells in the induction of DED was demonstrated by Chen et al. [18], which is entirely consistent with our findings of association of IFN-γ with tear production and TBUT but not being associated with the overall surface damage.

The measured tear IL-6 concentrations in our study correlate with the subjective complaints tear production and corneal damage, suggesting that the increased IL-6 in tear fluid may be a result of the progressing DED. Lam et al. [19] concluded that IL-6 correlated with the severity of symptoms and signs of tear dysfunctional syndrome, which is consistent with our findings. The exact source of IL-6 production in patients with DED is not known; however IL-6 can be produced by many cells including monocytes, macrophages and fibroblasts [20]. IL-6 is a potent cytokine and mediator in local and systemic inflammation and has effect on B cells and T cells [20-22]. IL-6 can also promote the differentiation of Th17 T cells, important regulators of autoimmune responses, which occur in DED and acute GvHD [21,23,24]. Much of our understanding of IL-6 and Th17 responses in GvHD come from mice models. Th17 cells, with the characteristic production of IL-17, can mediate pathology associated with GvHD in mice [24]. In multiple mouse strains blocking of IL-6 signaling dramatically attenuates GvHD and Th17 responses [11,25]. However Th17 responses may not be prerequisite for the development of GvHD [26]. It was suggested that posttransplantational epigenetic modifications of donor Th17 cells, which alter their cytokine production, lead to loss of IL-17 and increase in IFN-y producing cells [24]. In contrast to the mouse models, blocking of the IL-6 receptor in humans does not alter dendritic cell maturation, allogeneic T cell proliferation, or Th1/Th17 responses [27]. The role of IL-6 in ocular GvHD in humans may be beyond Th17 promoting and differ between mice models and human disease. Although a recent study with patients with dry eyes in aqueous-deficient patients suggested that elevated IL-6 levels in tear fluid had to be considered as a Th17 promoting response, the levels of IL-17 were not assessed [16]. We detect increased levels of IL-6 and IFN-γ in tear fluid of ocular GvHD, but were unable to detect IL-17 in any of the samples. It has been observed that IL-6 also promotes the differentiation of B cells and the induction of antibody production [28]. B cells play an important role in the development of GvHD which accounts for the effectiveness of B-cell depletion in the treatment of GVHD [29]. The role of IL-6 and the involvement of Th17 responses in human ocular GvHD remains to be elucidated.

In our study the group with ocular GvHD had a longer time interval between the measurements of cytokines than the group without ocular GvHD. This time difference can be explained by the fact that patients without ocular GvHD visited our OPD three months after SCT for screening purposes while patients with ocular GvHD visited our OPD for their treatment of ocular GvHD. However this time difference has no impact on the main message of this study. Confounders such as treatment with radiotherapy and chemotherapy and other medication were not taken in account due to the small number of patients.

Ocular GvHD is a serious ocular disorder significantly affecting the quality of life of patients following allo-SCT. The pathogenesis is unknown and the current therapy includes symptomatic regimens with lubricant and anti-inflammatory medications. To develop preventive and/or curative treatments the further clarification of pathogenesis of this disorder is necessary. In addition, ocular GvHD offers a unique opportunity to study the early onset of DED since it develops in many individuals after allo-SCT and the moment of onset is predictable. In contrast to former studies of DED, tear fluid analyses in ocular GvHD offer a unique opportunity for studying the pathogenesis of DED by determination of specific inflammatory mediators in diverse phases of this disorder. Giving the cross-sectional nature of this pilot study and its results, future prospective studies can concentrate on the changes occurring in tear fluid over time, before and after allo-SCT (in the preclinical stages of ocular GvHD and before the onset of DED) to identify the initial stages of ocular GvHD and determine the value of specific cytokines in the initiation and continuation of DED in ocular GvHD.

The results of this small study seem promising and show that IL-6 and IFN-γ were elevated in tear fluid of patients with ocular GvHD and correlated with different symptoms of dry eye disease.
